# Functional Genetic Frontiers in Plant ABC Transporters: Avenues Toward Cadmium Management

**DOI:** 10.3390/ijms262311662

**Published:** 2025-12-02

**Authors:** Deyvid Novaes Marques, Chase M. Mason

**Affiliations:** 1 Department of Genetics, Luiz de Queiroz College of Agriculture (ESALQ), University of São Paulo (USP), Piracicaba 13418-900, SP, Brazil; 2 Department of Biology, University of British Columbia Okanagan, Kelowna, BC V1W 5H9, Canada

**Keywords:** abiotic stresses, ABC transporters, cadmium, food safety, genetic engineering, heavy metal detoxification, plant biotechnology, phytoremediation, tolerance, transgenics

## Abstract

Cadmium (Cd) is a pervasive and highly toxic heavy metal that severely threatens environmental integrity, agricultural systems, plant metabolism, ecosystem health, and human food safety. Plants have evolved intricate detoxification mechanisms aimed at mitigating heavy metal toxicity, in which ATP-binding cassette (ABC) transporters play pivotal roles. This article contextualizes findings on the functional genetic manipulation of plant ABC transporters in Cd-exposed species, integrating evidence from model plants, crops, and transgenic systems. Key insights reveal how these transporters contribute to Cd distribution through multiple cellular and physiological pathways. We highlight the contribution of ABC transporters both in modulating Cd accumulation in plant tissues for food safety considerations and in regulating Cd-related parameters relevant to environmental cleanup and phytoremediation. Functional studies in different plant species demonstrate differential outcomes depending on transporter specificity and regulatory context. Cross-kingdom engineering further expands the biotechnological toolkit for Cd mitigation. Additionally, we performed a bibliometric analysis that underscores research trends linking ABC transporters with genetic manipulation strategies. The body of evidence highlights the perspective that precise modulation of ABC transporters—through strategies such as multi-gene engineering, tissue-specific expression, or fine-tuned regulatory approaches—offers a promising yet complex route to reconcile scientific and applied Cd management strategies.

## 1. Cadmium in Plants and the Strategic Targeting of ABC Transporters

Cadmium (Cd) is a highly toxic heavy metal and a significant abiotic stress factor, representing a major pollutant in agricultural environments and posing serious threats to both plant metabolism and food safety. Its phytotoxic effects manifest through multiple physiological disruptions in plants, with the severity of toxicity varying depending on concentration, plant species, and growth conditions (as we previously discussed [1]). Once absorbed by the roots, Cd^2+^ is potentially immobilized in cell walls and vacuoles, while another fraction is translocated through the xylem and, to some extent, via the phloem. This uneven distribution and compartmentalization are crucial for the plant’s ability to regulate Cd homeostasis and enhance tolerance [2]. To cope with Cd toxicity, plants have evolved complex defense mechanisms, including metal chelation, active efflux, and subcellular sequestration [3].

ATP-binding cassette (ABC) transporters are integral membrane proteins that actively move diverse substrates, including heavy metals, across cellular membranes. These molecules are ubiquitous across multiple living organisms, from bacteria to higher plants and animals. In eukaryotes, ABC transporters are phylogenetically classified into eight subfamilies, designated ABCA to ABCH [4]. They are present in various plant species, with the number of ABC transporter genes differing among species [5]. For example, this family includes over 120 genes in species like *A. thaliana* and rice (*Oryza sativa*), and these proteins are fundamentally involved in numerous physiological functions, including adaptation and resilience to abiotic stresses [6].

Structurally, ABC proteins share a conserved modular organization composed of two core elements: a hydrophobic transmembrane domain typically formed by six α-helices, and a cytosolic nucleotide-binding domain responsible for ATP binding [7]. Historically, it was believed that ABC importers were exclusive to prokaryotes and archaea, whereas eukaryotic ABCs functioned solely as exporters. However, some authors have highlighted that certain plant ABC transporters can also exhibit importer activity [8]. Furthermore, functional analyses have revealed the involvement of specific ABC transporters in metal transport and detoxification processes. For instance, TaABCG2-5B in wheat has been identified as an importer important for Cd^2+^ uptake and transport [9]. In *Arabidopsis*, AtABCC3 mediates vacuolar sequestration of phytochelatin (PC)–Cd complexes, contributing to Cd detoxification [7]. Moreover, in such a plant model, the ABC transporter AtATM3 has been implicated in conferring resistance to Cd(II) and lead (Pb)(II), likely by facilitating the mitochondrial transport of Cd–thiol conjugates (GSH-related) and/or Fe–S clusters [10].

It becomes clear that investigating ABC transporters through functional genetic approaches is relevant for moving from descriptive understanding toward practical applications in Cd mitigation. Although previous studies have provided valuable insights into certain aspects of ABC transporters in Cd-exposed plants, many findings remain descriptive or correlative, leaving mechanistic understanding and functional applications largely unexplored. Functional genetic manipulation in plants enables direct testing of gene function, precise modulation of transporter activity, and assessment of phenotypic consequences, which may result in differential outcomes. This approach provides a more mechanistic and translational perspective, highlighting the need for holistic evaluation in this research area.

Despite its considerable potential, research on functional genetic manipulation and targeted genetic engineering of ABC transporters remains relatively scarce across multiple plant species and experimental contexts. Importantly, no article has offered an integrated overview and presented the current panorama specifically addressing the functional genetic manipulation of ABC transporters in Cd-exposed plants. Ongoing studies indicate that ABC transporters play a role in modulating both food safety and plant phytoremediation potential, with effects that can be highly context-dependent. In this article, we focus on the functional genetic manipulation of ABC transporters in Cd-exposed plants. Additionally, we further enriched this perspective by conducting a bibliometric analysis, which is discussed throughout the manuscript and based on international databases focusing on publications within this research field. By presenting the following sections, we aim to inspire further research and provide an integrated overview of the current landscape and emerging perspectives in this important scientific field. Ongoing studies are highlighted with emphasis on their relevance and translational potential—particularly in plant models, crop species, and potential phytoremediators with direct implications for sustainable agriculture—alongside nuanced perspectives of the context-dependent outcomes of functional genetic manipulation.

**Figure 1:**
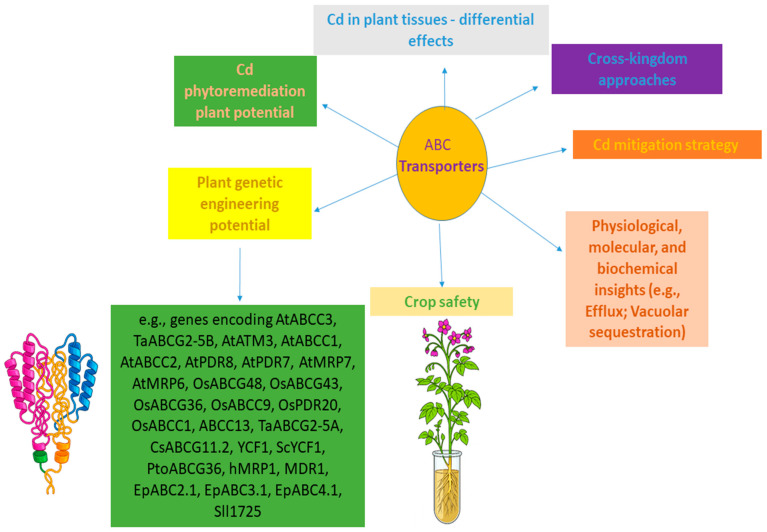
Graphical overview of selected topics in research on ABC transporters, Cd management, and functional genetic manipulation in plants.

This article consolidates these dimensions regarding functional genetic manipulation within the plant Cd research context. This review is structured to provide a clear, integrative progression—from plant systems to crop-level responses, phytoremediation-related information, and multiple agricultural perspectives—together with an international bibliometric analysis, thereby offering a relevant and forward-looking overview in the fields of plant Cd management, functional genetic manipulation, and ABC transporter biology. A graphical overview of topics related to the research field is presented in Figure 1, and specific details are further elaborated throughout the article’s thematic sections.

## 2. Functional Genetic Manipulation Focusing on ABC Transporters in Plant Models

The meticulous dissection of Cd detoxification mechanisms in the model plant *A. thaliana* has revealed a complex interplay of ABC transporters operating across various subcellular compartments. These transporters represent critical targets for genetic modification aimed at enhancing plant resilience to heavy metal stress and optimizing phytoremediation strategies.

Vacuolar sequestration stands as a primary defense mechanism against Cd toxicity. To better integrate these mechanisms, the following evidence collectively illustrates how distinct ABCC transporters operate within the context of key plant model systems to establish a coordinated framework for Cd mitigation. Brunetti et al. [7] demonstrated that the ABCC transporter AtABCC3 in *A. thaliana* is fundamentally involved in the vacuolar transport of PC–Cd complexes. Knockout mutants for the *AtABCC3* gene (*abcc3*) in *A. thaliana* seedlings demonstrated heightened sensitivity to Cd, whereas seedlings overexpressing *AtABCC3* (*AtABCC3ox*) showed increased tolerance; this phenotype directly correlated with cellular localization analyses in leaf protoplasts, revealing decreased vacuolar Cd accumulation in *abcc3* and increased vacuolar Cd in *AtABCC3ox* plants. Crucially, AtABCC3 activity is strictly PC-dependent, as its overexpression did not alleviate Cd toxicity in PC-deficient lines. Furthermore, *AtABCC3* overexpression effectively complemented the Cd sensitivity of *atabcc1 atabcc2* double mutants, which are deficient in *AtABCC1* and *AtABCC2*, highlighting a significant compensatory role. The expression of *AtABCC3* is regulated by Cd levels, showing higher transcript levels at elevated Cd concentrations and a notable increase in *atabcc1 atabcc2* mutants, indicative of an intricate regulatory network ensuring detoxification efficiency [7]. Complementing these findings, Park et al. [11] further investigated the role of AtABCC1 and AtABCC2 as crucial vacuolar transporters for PC–Cd(II) and PC–Hg(II) complexes. *atabcc1* single or *atabcc1 atabcc2* double knockout mutants exhibited an increased shoot-to-root ratio of Cd as well as hypersensitivity to both Cd(II) and Hg(II), with Cd predominantly localized in the cytosol of mutant protoplasts compared to the vacuole in wild-type (WT) cells. Overexpression of *AtABCC1* in *Arabidopsis* enhanced both Cd(II) tolerance and accumulation, underscoring the practical utility of these transporters for potential phytoremediation applications. Within this broader research topic, integrating the functional roles of AtABCC1, AtABCC2, and AtABCC3 highlights how vacuolar PC–metal sequestration constitutes a unified, model-system-informed strategy for effective Cd mitigation in plants. However, the exact hierarchy and the extent to which ABC proteins such as AtABCC1 and AtABCC2 function as transporters for Cd and Hg—given their related role in other heavy metal contexts, such as arsenic (As) detoxification [11]—remain unclear and warrant further investigation.

Beyond vacuolar storage, other ABC-related studies also considered other cellular compartments which also contribute to Cd homeostasis. Together, these findings indicate that Cd tolerance arises from a coordinated action across multiple organelles, rather than from isolated subcellular events, thereby reinforcing the need to interpret each transporter within an integrated cellular network. A distinct role was uncovered for AtATM3, an ABC transporter localized in the mitochondria, in Cd resistance in *A. thaliana*. In this context, transitioning from vacuolar sequestration to mitochondrial regulation highlights how different ABC systems sequentially shape cellular detoxification pathways. *AtATM3* was shown to be upregulated in roots upon Cd treatment [10]. Overexpression of *AtATM3* conferred enhanced Cd resistance, while *atatm3* mutants were more sensitive. Intriguingly, *AtATM3*-overexpressing plants exhibited increased Cd content in the shoot, a highly desirable trait for phytoremediation. The authors hypothesized that the heightened (Cd(II)) accumulation in the shoot of *A. thaliana* results from AtATM3 mediating the export of Cd–thiol (GSH-related) complexes across the mitochondrial membrane. This mechanism was proposed to influence cytosolic GSH levels, possibly promoting increased long-distance Cd translocation from root to shoot, a characteristic deemed beneficial for phytoremediation purposes [10], although the precise molecular mechanisms governing this enhanced translocation warrant deeper investigation. In addition, this shift from organelle-specific functions to whole-plant outcomes may exemplify how subcellular mechanisms collectively contribute to long-distance Cd transport and overall homeostasis.

Within the context of plasma membrane, which also plays a vital role in preventing Cd accumulation through active efflux, some authors have identified the ABC transporter AtPDR8, found more prominently in root hair and epidermal cells, as a major plasma membrane efflux pump for Cd^2+^ or Cd conjugates in *A. thaliana* [12]. Thus, in addition to the mitochondrial mechanisms presented previously, this aspect regarding plasma membrane-mediated detoxification underscores how distinct ABC transporters operate in a sequential and/or compartmentalized manner to maintain overall Cd homeostasis. *AtPDR8* expression is significantly induced by Cd treatment. Plants overexpressing *AtPDR8* showed enhanced Cd resistance and reduced Cd content, while RNAi transgenic plants and T-DNA insertion lines were markedly more sensitive and accumulated higher Cd levels. AtPDR8 localization to the plasma membrane and its direct promotion of Cd extrusion in protoplast flux assays strongly support its role in active cellular Cd exclusion [12]. This functional specificity is further emphasized by the observation that AtPDR7, a closely related homolog, showed only a marginal and statistically insignificant impact on Cd sensitivity when silenced, suggesting AtPDR8 is indeed the primary Cd efflux pump among these paralogs [12]. This progression from subcellular detoxification to cell-surface efflux exemplifies how plants integrate organelle-specific and membrane-level responses into a unified Cd exclusion strategy.

Furthermore, some findings pointed out that the complex control over Cd distribution and long-distance translocation involves transporters with potentially dual localization. Following the shift from plasma membrane efflux systems to more integrative mechanisms, these observations reinforce that ABC transporter-related Cd homeostasis relies on multilayered interactions across distinct cellular compartments. The heterologous expression of ABC transporter gene *AtMRP7* from *A. thaliana* in *Nicotiana tabacum* (tobacco) significantly modified Cd accumulation, distribution, and tolerance. It was observed that AtMRP7 is localized to both the tonoplast and the plasma membrane in tobacco cells [13]. Its overexpression enhanced Cd tolerance and increased Cd concentration in leaf vacuoles, indicating improved vacuolar storage, while also promoting more efficient Cd retention in roots, possibly influencing root-to-shoot translocation. This dual-targeting mechanism exemplifies how ABC transporters may integrate organelle-level sequestration with membrane-associated retention aspects to fine-tune long-distance Cd fluxes. The distinct patterns of ABC-related Cd accumulation observed at varying metal concentrations suggest a nuanced, concentration-sensing mechanism that regulates detoxification and long-distance transport [13].

Transitioning from dual-localized transporters to genotype or plant-specific loss-of-function evidence highlights how individual ABC members may contribute differently to Cd management, with some exerting effects that are not directly mirrored in bulk metal or thiol levels. Some authors in turn found that ABC transporter *Atmrp6* knockout mutants of *A. thaliana* exhibited increased Cd sensitivity, notably affecting leaf development, despite displaying comparable root elongation and ramification to WT plants [14]. Interestingly, this increased sensitivity was not consistently accompanied by significant changes in bulk Cd, GSH, γ-glutamylcysteine (γ-EC), or PC contents across different experimental setups [14]. This suggests that AtMRP6 might be involved in subtler aspects of Cd response, possibly related to specific developmental processes, the transport of specific Cd-bound compounds beyond bulk PCs, or localized effects not reflected in overall tissue content. Taken together, these findings illustrate that while some ABC transporters exert clear biochemical impacts, others may influence Cd tolerance through more specialized or context-dependent functions, and the authors critically posited the possibility of functional redundancy within the AtMRP3/AtMRP6/AtMRP7 cluster, a hypothesis that demands further exploration to fully elucidate the contribution of each member to Cd homeostasis [14].

**Figure 2:**
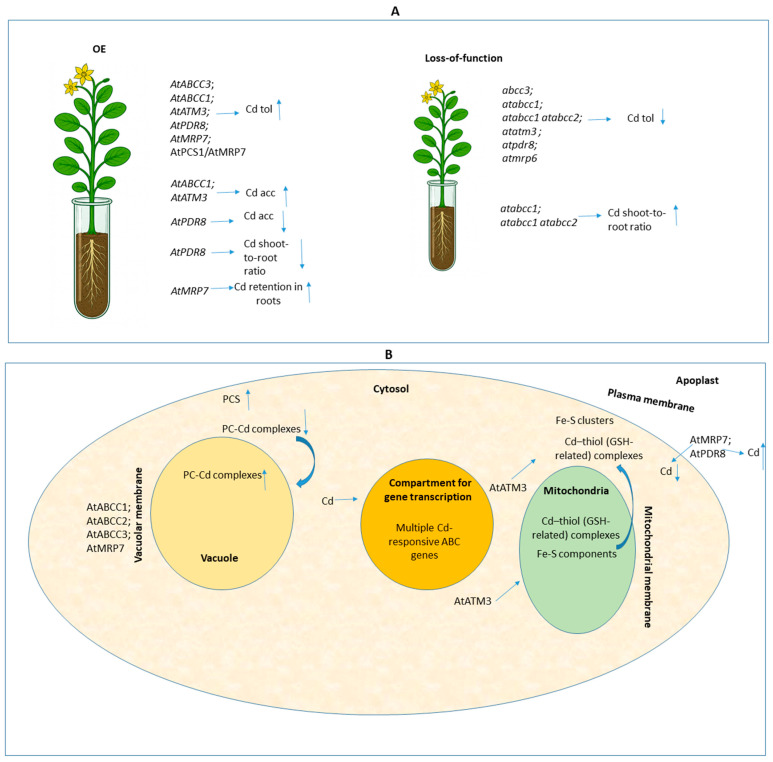
Schematic representation of integrated research overview covering key exemplary topics from functional genetic manipulation studies focusing on ABC transporters and Cd exposure in plant model systems. Upward (↑) and downward (↓) arrows indicate increased and reduced ABC gene expression, respectively, as well as increases (↑) or reductions (↓) in Cd accumulation, uptake, Cd tolerance, or other aspects regarding mechanism-specific responses described in each study. The other arrows represent the conceptual direction of transport or induction or the downstream effect generated. The semicolon (;) is used to separate different genetic backgrounds—mutant lines, overexpression lines, or any genotypes that share a common functional outcome or shared feature. Slash-grouped terms (/) correspond to co-expressed genes. Panels (**A**,**B**) illustrate the information from the referenced studies considered in Section 2 at the whole-plant and cellular levels, respectively. The following abbreviations were used in the figure: Acc: accumulation; Cd: cadmium; GSH: glutathione; OE: overexpression; PC: phytochelatin; PCS: phytochelatin synthase; Tol: tolerance.

An integrated overview of such functional-genetic studies using plant model systems (Figure 2) reveals that ABC transporters do not follow a single, uniform pattern in Cd handling, and this divergence is central for interpreting their biotechnological potential—particularly when considering translational goals in other plant species, whether aimed at food safety or at phytoremediation for soil decontamination.

While some transporters enhance tolerance without altering bulk Cd content—as reported for AtABCC3, whose vacuolar sequestration capacity mitigates toxicity without increasing whole-plant accumulation [7]—others clearly link detoxification to greater metal loading. This is evident, for instance, in the overexpression of AtABCC1, which simultaneously boosts Cd tolerance and total accumulation across organs [11], and in the mitochondrial exporter AtATM3, which enhances Cd accumulation in shoots and potentially increases biomass-harvestable Cd [10]. Conversely, distinct physiological aspects arise in exclusion-type mechanisms such as AtPDR8, which improves tolerance by lowering Cd levels via efflux at the plasma membrane [12]. Additional layers of complexity emerge when increased sensitivity occurs despite unchanged tissue Cd concentrations, as observed in Atmrp6 mutants, indicating that localized transport of specific Cd conjugates can drive physiological outcomes independently of bulk metal load [14]. Such contrasting findings—ranging from modulated uptake coupled to tolerance (e.g., AtMRP7 in tobacco; [13]) to tolerance without necessarily altering Cd accumulation or even exclusion—underscore that engineering ABC transporters for phytoremediation or crop protection requires context-dependent choices. Also importantly, these discrepancies highlight the need for careful translational pathways that connect controlled laboratory phenotypes to field performance, where soil chemistry, rhizosphere interactions, and agronomic constraints can shift the balance between desirable metal uptake and unintended accumulation.

The collective research on genetically modified *A. thaliana* and plant model-related knowledge further highlights the multifaceted nature of Cd detoxification and homeostasis, encompassing vacuolar sequestration, mitochondrial processing, and plasma membrane efflux, orchestrated by diverse ABC transporters. While the critical role of PC-Cd complex transport by AtABCC1, AtABCC2, and AtABCC3 was discussed, the distinct functions of transporters like mitochondrial AtATM3 in influencing shoot accumulation and plasma membrane AtPDR8 in cellular extrusion underscore a highly sophisticated and interconnected network. Ambiguities surrounding the exact mechanism of AtMRP6-related sensitivity or the dual localization of AtMRP7 with its concentration-dependent effects underscore the remaining complexities. Future research might prioritize a comprehensive understanding of the precise molecular substrates and interaction dynamics of each ABC transporter, particularly for those with less defined roles or broad substrate specificities. Elucidating the hierarchy, compensatory mechanisms, and regulatory interplay within gene clusters, such as the AtMRP3/AtMRP6/AtMRP7 cluster, is paramount. Furthermore, it is critical to definitively establish whether AtABCC1/AtABCC2 and AtABCC3 are the main transporters for Cd and Hg in comparison to As, as well as other relevant heavy metal-related comparisons, and how environmental Cd concentrations precisely modulate the expression and activity of these transporters.

## 3. Harnessing ABC Transporters for Cadmium Management in Crops

The information obtained from studies in plant models (such as *Arabidopsis*) regarding ABC transporter function, as presented in the previous section, offers immense potential for genetic engineering to bolster heavy metal tolerance and accumulation in a broader spectrum of plant species, including economically important crops. Indeed, the engineering of ABC proteins in major crops represents a relevant strategy for Cd mitigation, addressing both plant health and food safety concerns in contaminated environments. However, the practical implementation of such genetic modifications necessitates a thorough evaluation of potential pleiotropic effects and a careful balance between reducing Cd accumulation and maintaining, or even enhancing, overall crop performance and safety.

In rice, several ABC transporters have been investigated for their roles in Cd homeostasis and distinct effects have been reported. For example, heterologous expression of *OsABCG48* in *Schizosaccharomyces pombe*, *A. thaliana*, and rice conferred enhanced Cd tolerance, while its overexpression in rice reduced root Cd accumulation and promoted lateral root development. These findings highlight OsABCG48 as a promising target for developing low-Cd rice cultivars, complementing strategies by showcasing the intrinsic potential of plant ABC transporters for safer crop bioengineering. It is worth mentioning that, according to a transmembrane analysis, OsABCG48 displays the typical architecture of full-size G-type ABC transporters, containing two conserved transmembrane domains and likely mediating substrate translocation across the lipid bilayer [15]. Taken together, OsABCG48 exemplifies a rice ABC transporter that may mitigate Cd toxicity primarily through limiting root uptake, providing a functional benchmark for comparison with other rice transporters that instead enhance Cd influx or sequestration. Thus, further studies are relevant to elucidate such precise transport mechanisms and substrate specificity in planta.

Conversely, overexpression of *OsABCG43*, identified as a Cd importer, strikingly led to increased Cd accumulation in rice, causing phytotoxicity, retarded plant growth, accelerated senescence, and enhanced sensitivity to Cd stress. Importantly, this also adversely affected the proliferation of leaf bacteria and impaired bacterial virulence factors [4], highlighting impacts on aspects regarding plant–microbe interactions. The G-type ABC transporter OsABCG36 in rice in turn has been highlighted to be involved in Cd tolerance by facilitating Cd efflux from root cells. Knockout of *OsABCG36* resulted in increased Cd accumulation in root cell sap and enhanced Cd sensitivity, without affecting Cd accumulation in the shoots, suggesting its crucial role in protecting root cells from Cd toxicity [16]. In another investigation, OsABCC9, a C-type ABC transporter localized at the tonoplast, was suggested to contribute to Cd sequestration into root vacuoles in rice, potentially by mediating the transport of Cd or Cd–thiol complexes. *OsABCC9* knockout mutants exhibited increased sensitivity to Cd and accumulated higher Cd levels in roots and shoots, and critically, in the grain and xylem sap, highlighting its importance for limiting Cd transfer to edible parts for safety [17]. Another recent study investigated OsPDR20, an ABCG family transporter which is localized to the plasma membrane in rice, as being involved in Cd accumulation and homeostasis. While its knockdown by RNA interference led to compromised growth and moderately attenuated root/shoot elongation under Cd stress, it resulted in higher Cd accumulation in rice straw (lower leaves and culm) and grain in field trials [18]. This increased Cd accumulation in edible parts due to *OsPDR20* suppression poses a significant food safety aspect for grain consumption, underscoring that modifications aimed at understanding gene function can have detrimental practical consequences if not carefully managed, although it suggests potential for phytoremediation [18]. These transporters illustrate contrasting functional strategies within rice—ranging from Cd import to efflux and vacuolar sequestration, as well as modulation of whole-plant Cd partitioning—thereby underscoring how individual ABC genes can drive markedly different physiological and agronomic outcomes.

Altogether, these findings reveal that engineering ABC proteins in rice is far from straightforward. Their pleiotropic and occasionally paradoxical effects demand a nuanced approach that integrates multiple dimensions, including transporter specificity, plant–microbe interactions, and food safety considerations, into future bioengineering strategies. Within this context, the contrasting outcomes further underscore the complexity of manipulating ABC transporters, as subtle differences in substrate specificity, tissue localization, and regulatory networks can determine whether genetic interventions result in beneficial detoxification or unintended toxicity. Therefore, a more predictive, systems-level understanding of transporter cross-talk and metal homeostasis is crucial before implementing such modifications in breeding programs or Cd mitigation-related applications. A promising approach for mitigating multiple toxic elements in rice involved the co-overexpression of *OsPCS1*, *OsABCC1*, and *OsHMA3*. This synergistic strategy effectively decreased As and Cd concentrations in the grain by 92.1% and 98%, respectively, compared with non-transgenic rice plants, without causing any defects in plant growth, reproduction, or the content of essential mineral nutrients in the grain [19]. This tripartite co-overexpression represents an effective and safe approach for developing low-As and low-Cd rice, demonstrating the potential for overcoming negative pleiotropic effects through combined genetic strategies [19]. It is important to note that while OsABCC1 is critical for As detoxification, it was observed that its expression does not appear to affect Cd tolerance, demonstrating the specificity of certain ABC proteins [20]. This finding highlights that although OsABCC1 alone does not confer Cd tolerance [20], its co-overexpression with *OsPCS1* and *OsHMA3* might synergistically mitigate both As and Cd toxicity [19], emphasizing the value of multi-gene strategies for enhancing tolerance to multiple toxic elements. In this broader comparative view, multi-gene stacking emerges as a strategically distinct alternative to single-gene modifications, offering a way to reconcile transporter-specific limitations and achieve more consistent management of toxic elements across plant physiological compartments.

In the wheat (*Triticum aestivum*) research context, some authors have also highlighted that phytic acid, synthesized in the plant cytosol and transported into vacuoles by ABC multidrug resistance-associated transporters, is a major seed phosphate reserve that chelates essential micronutrients and reduces their bioavailability [21]. RNAi-mediated silencing of *ABCC13* reduced phytic acid content in mature wheat grains, a desired trait for nutritional improvement, but concurrently caused developmental defects during grain filling and increased sensitivity to Cd in transgenic roots, along with overall reduced Cd accumulation, impaired lateral root development under Cd exposure, and defects in metal uptake [21]. These findings underscore the complex interplay of physiological functions and highlight the critical need to carefully evaluate genetic modifications to modulate specific effects on crop yield and stress tolerance, even when pursuing beneficial traits such as low phytic acid, as previously reported [21]. Together, these outcomes illustrate how altering a single vacuolar ABC transporter in wheat can generate broad physiological trade-offs, contrasting with crop studies where certain ABC transporters confer more direct detoxification benefits, thereby emphasizing species-specific constraints when, for instance, targeting phytic acid or Cd pathways.

More recently, the G-type ABC transporters TaABCG2-5B and its homolog TaABCG2-5A have been investigated through loss-of-function mutants and stable overexpression lines in wheat (*Triticum turgidum* subsp. *durum*). Functional analysis revealed that TaABCG2-5B acts as a plasma membrane importer, facilitating the transport of both salicylic acid (SA) into the cytoplasm and Cd^2+^ uptake, in parallel with plant immunity against Fusarium head blight (FHB). Conversely, the homologous gene *TaABCG2-5A* also promoted Cd^2+^ uptake in wheat roots—as its mutant lines showed an approximately 30% decrease in Cd^2+^ accumulation—but did not significantly influence SA accumulation or FHB resistance [9]. This selective functional differentiation, together with natural sequence variations in *TaABCG2* among wheat varieties, makes such a gene family a valuable target for breeding wheat cultivars with reduced Cd^2+^ accumulation [9] without compromising essential SA-mediated defense mechanisms. These findings open new perspectives for the field of plant Cd research, suggesting that ABC transporter engineering can move beyond detoxification-based strategies toward precision modulation of multifunctional transporters that integrate heavy metal homeostasis with hormonal and immune signaling. Future efforts may focus on allelic fine-tuning or domain-specific editing of ABC transporters such as TaABCG2 members to decouple Cd transport from defense-related functions, thereby designing crop genotypes that achieve low-Cd accumulation, enhanced resilience, and optimized metabolic balance under field conditions. In a broader comparative perspective, the divergent roles of *TaABCG2* paralogs—spanning different aspects, including Cd uptake, hormonal transport, and immune regulation—may contrast with the more specialized functions observed in rice ABC transporters, reinforcing the notion that wheat ABC families may require precision, gene-by-gene tailoring to avoid compromising immunity while reducing Cd accumulation.

In tea plants (*Camellia sinensis*), a recently published study indicated that the ABC transporter CsABCG11.2 exhibits a dual role in Cd dynamics and amino acid metabolism. CsABCG11.2 may facilitate Cd uptake and translocation from roots to young shoots, increasing metal accumulation and sensitivity in aerial tissues, while Thea mitigated Cd-induced oxidative stress and improved photosynthetic performance [8]. Gene knockdown experiments in tea demonstrated that reducing *CsABCG11.2* expression significantly lowered Cd levels in young leaves, while overexpression in *Arabidopsis* led to enhanced Cd sensitivity and higher shoot accumulation [8]. Conversely, CsABCG11.2 also mediates the transport of the beneficial amino acid theanine (Thea), which competes with Cd for uptake and alleviates Cd-induced toxicity. Heterologous yeast assays confirmed this competitive transport, and exogenous Thea application mitigated Cd-induced growth inhibition and chlorosis, particularly in hypersensitive overexpression lines. Moreover, Thea reversed Cd-induced suppression of nitrogen assimilation and caffeine biosynthesis, linking its protective effect to broader metabolic regulation [8]. These findings suggest that although CsABCG11.2 contributes to enhanced Cd concentration, its role in Thea transport provides an indirect protective effect. For breeding low-Cd tea, it was highlighted that instead of complete knockout, selecting *CsABCG11.2* alleles with enhanced affinity for Thea or reduced Cd transport could optimize the balance between maintaining beneficial metabolite production and minimizing Cd accumulation [8]. Thus, future research might move beyond single-*ABC* gene manipulation to strategically design tea cultivars with optimized Thea metabolism, coordinated nutrient use efficiency, and controlled Cd accumulation. Such efforts might also assess potential trade-offs between metabolic benefits, stress resilience, research focused on cross-species plant comparisons, and commercial quality, ensuring that genetic or biochemical modulation enhances both plant health and market value rather than introducing unintended drawbacks.

**Figure 3:**
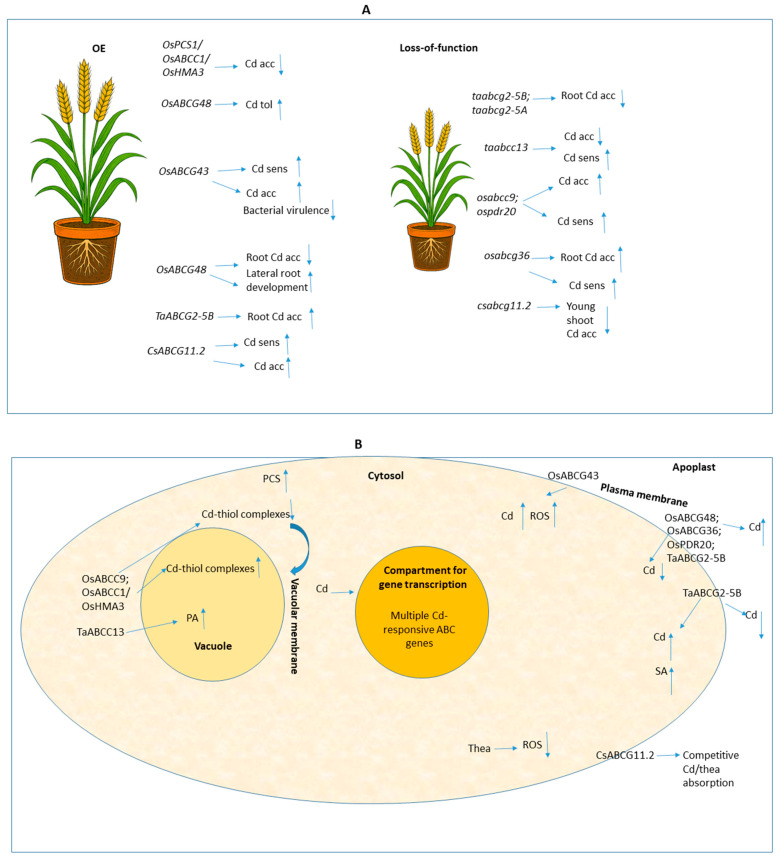
Schematic representation of integrated research overview covering key exemplary topics from functional genetic manipulation studies focusing on ABC transporters and Cd exposure within plant crop context. Upward (↑) and downward (↓) arrows indicate increased and reduced ABC gene expression, respectively, as well as increases (↑) or reductions (↓) in Cd accumulation, uptake, Cd tolerance, or other aspects regarding mechanism-specific responses described in each study. The other arrows represent the conceptual direction of transport or induction or the downstream effect generated. The semicolon (;) is used to separate different genetic backgrounds—mutant lines, overexpression lines, or any genotypes that share a common functional outcome or shared feature. Slash-grouped terms (/) correspond to co-expressed genes. Panels (**A**,**B**) illustrate the information from the referenced studies considered in Section 3 at the whole-plant and cellular levels, respectively. The following abbreviations were used in the figure: Acc: accumulation; Cd: cadmium; OE: overexpression; PA: Phytic acid; PCS: phytochelatin synthase; ROS: reactive oxygen species; Sens: sensitivity; Thea: theanine; Tol: tolerance.

The set of functional genetic studies in crop plant research shows that ABC transporters shape Cd responses through mechanisms that may be convergent, divergent, or even contradictory when examined across developmental stages, genotypes, environmental conditions, and experimental contexts (Figure 3).

Clear oppositions emerge between exclusion-based strategies—such as OsABCG48, whose gene overexpression in rice markedly reduced root Cd accumulation while enhancing tolerance and promoting lateral root formation [15], or OsABCG36, whose loss-of-function leads to elevated Cd retention and pronounced root sensitivity [16]. Still, other cases illustrate that increased uptake can be detrimental: the vascular importer OsABCG43 drives disproportionately high Cd loading and leads to significant phytotoxicity, accelerated senescence, and reduced growth in rice, despite its antibacterial side effects, which may confer context-dependent effects within particular pathosystems [4]. Silencing *OsPDR20* also disrupts Cd distribution at maturity, causing substantial Cd enrichment in culm and, critically, in grains—up to nearly threefold increases—thereby posing major food-safety risks despite sometimes reducing Cd in select tissues such as upper leaves [18]. Wheat transporters also illustrate that lower Cd accumulation does not necessarily equate to enhanced tolerance: knockdown of *TaABCC13* reduces total Cd but still induces severe sensitivity and developmental defects, underscoring the essential role of localized detoxification processes [21]. Conversely, reducing Cd uptake can introduce trade-offs that compromise agronomic stability, exemplified by TaABCG2-5B, whose mutation lowers Cd entry but simultaneously disrupts salicylic-acid-dependent immunity, resulting in heightened susceptibility to Fusarium head blight and yield penalties [9]. Even transporters with dual functions may complicate engineering strategies, as shown for CsABCG11.2, which increases Cd accumulation and sensitivity in *Arabidopsis* yet also mediates uptake of Thea, an endogenous metabolite capable of competitively limiting Cd transport and alleviating toxicity [8]. Taken together, these contrasting outcomes—positive or negative depending on whether the trait is evaluated for food-crop safety, pathogen resistance, field growth, or other plant-related aspect—demonstrate that translating transporter-based genetic modifications from laboratory assays to field applications requires carefully weighing exclusion versus sequestration goals, anticipating pleiotropic constraints, and integrating soil chemistry, microbial interactions, and tissue-specific allocation patterns into the decision-making framework within the ABC research context.

The studies involving plant crop research collectively demonstrate the critical and often multifaceted roles of ABC transporters in modulating Cd accumulation and tolerance in major crops. However, studies across multiple crop species remain limited in the context of plant genetic engineering and therefore require further expansion and functional validation. Emerging evidence indicates that manipulating specific ABC transporter isoforms could become a promising strategy for Cd mitigation aspects. However, while genetic modifications targeting these transporters offer promising avenues for Cd mitigation, they also emphatically highlight the importance of thoroughly assessing potential pleiotropic effects on plant growth, yield, essential nutrient content, and overall crop safety. Strategies that achieve substantial reduction in grain Cd content without compromising other vital plant functions, or even improving them, such as the co-overexpression approach in rice [19], represent optimal solutions for developing safer and more resilient crops in Cd-contaminated agricultural systems. This comprehensive evaluation is paramount for translating genetic insights into practical, safe, and agronomically sound solutions or related strategies.

## 4. Functional Manipulation of ABC Transporters Toward Cadmium Phytoremediation

The intricate roles of ABC transporters also present a promising avenue for modulating Cd phytoremediation capabilities through functional genetic manipulation. Various strategies utilizing ABC transporters have demonstrated success in changing parameters that are related to both tolerance and accumulation of Cd in different plant species. A primary strategy to address this aspect involves enhancing vacuolar sequestration of Cd. For instance, the yeast cadmium factor 1 (YCF1), a vacuolar ABC transporter from *Saccharomyces cerevisiae*, has been successfully introduced into *Brassica juncea* (Indian mustard), a heavy metal accumulator plant species suitable for phytoremediation [22]. Transgenic *B. juncea* plants overexpressing *YCF1* exhibited enhanced tolerance to Cd(II) stress, showing a 1.3- to 1.6-fold increase compared to WT plants. Crucially, the shoot tissues of these transgenic seedlings accumulated approximately 1.5- to 2-fold higher Cd(II) levels, indicating significantly increased heavy metal accumulation. This enhanced accumulation is attributed to YCF1’s role at the vacuolar membrane, actively translocating heavy metals conjugated to GSH from the cytoplasm into vacuoles, sequestering Cd as Cd(II)-GSH [22]. Similarly, genetic engineering of poplar plants, which offer rapid growth and high biomass essential for phytoremediation, with *ScYcf1* and *ScYHL035C* (genes encoding multidrug resistance-associated protein (MRP) subfamily ABC transporters), has led to highly improved resistance to heavy metals, including Cd and Pb [23]. Specifically, a sterile line of *Populus alba × P. tremula* var. *glandulosa* transformed with *ScYCF1* demonstrated enhanced growth, reduced toxicity symptoms, and increased Cd content in aerial tissues compared to non-transgenic plants when tested for phytoremediation aspects in mine tailing soil. These transgenic poplars also accumulated increased amounts of Cd, Zn, and Pb in their roots, establishing an extensive root system. This enhancement is consistent with the established mechanism of ScYCF1, which potentially facilitates Cd detoxification by pumping GSH-conjugated Cd into the vacuole, as previously highlighted [24].

Another significant focus lies on ABC transporters localized to mitochondria or the plasma membrane, impacting heavy metal homeostasis and efflux. The *A. thaliana* AtATM3, an ABC transporter located at the mitochondrial membrane and involved in Fe–S cluster biogenesis, has been utilized to improve heavy metal tolerance and accumulation in *B. juncea* [25]. Overexpression of *AtATM3* in *B. juncea* led to enhanced tolerance to Cd(II) and Pb(II) stresses, with shoot tissues accumulating 1.5- to 2.5-fold higher levels of these metals than WT seedlings. This enhanced capacity was linked to higher expression levels of *BjGSHII* (*B. juncea* glutathione synthetase II) and *BjPCS1* (encoding a PC synthase) induced by *AtATM3* overexpression, along with the regulation of several other metal transporters. Furthermore, *AtATM3* transgenic *B. juncea* displayed approximately 1.5-fold higher biomasses than WT plants, a beneficial characteristic for phytoremediation [25]. More recently, *AtATM3* was introduced into *Brassica napus* cv. BARI Sarisha-8, a genotype highly resistant to Cd and Pb stresses. Transgenic *B. napus* expressing *AtATM3* showed notable improvements in Cd tolerance, with a 1.4- to 1.7-fold increase in fresh weight compared to the WT counterpart [26]. In *Populus tomentosa*, a suited species for tackling soil contamination and phytoremediation, the ABC transporter gene *PtoABCG36* was isolated and found to be expressed in leaves, stems, and roots, with expression strongly induced under Cd stress. Subcellular localization analyses revealed its presence at the plasma membrane, as confirmed through transient expression assays in tobacco. Functional characterization in yeast and *Arabidopsis* demonstrated that *PtoABCG36* overexpression reduced Cd accumulation and enhanced tolerance by actively exporting Cd^2+^ out of cells, functioning as an efficient Cd extrusion pump [27].

**Figure 4:**
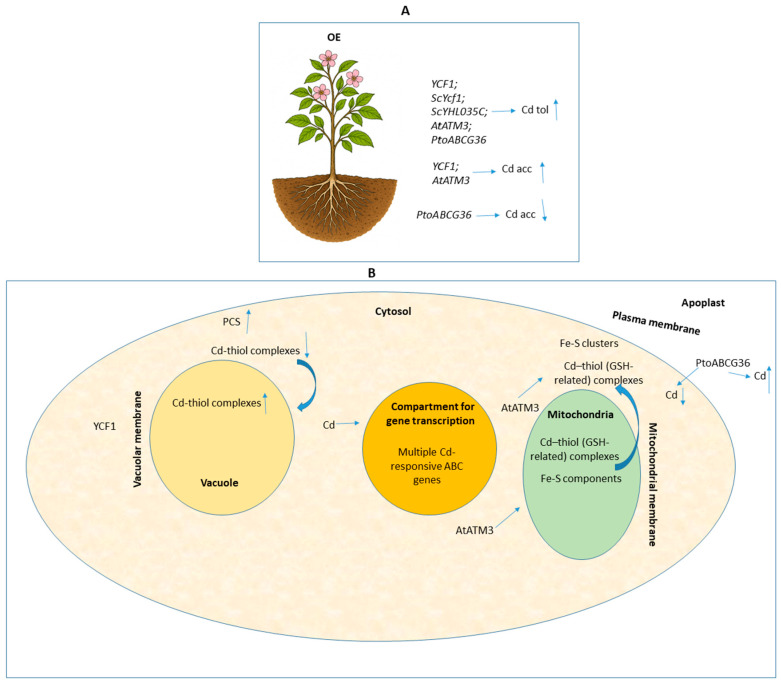
Schematic representation of integrated research in plants with phytoremediation potential, highlighting key topics from functional genetic manipulation studies on ABC transporters and Cd exposure. Upward (↑) and downward (↓) arrows indicate increased and reduced ABC gene expression, respectively, as well as increases (↑) or reductions (↓) in Cd accumulation, uptake, Cd tolerance, or other aspects regarding mechanism-specific responses described in each study. The other arrows represent the conceptual direction of transport or induction or the downstream effect generated. The semicolon (;) is used to separate different genetic backgrounds—mutant lines, overexpression lines, or any genotypes that share a common functional outcome or shared feature. Panels (**A**,**B**) illustrate the information from the referenced studies considered in Section 4 at the whole-plant and cellular levels, respectively. The following abbreviations were used in the figure: Acc: accumulation; Cd: cadmium; GSH: glutathione; OE: overexpression; PCS: phytochelatin synthase; Tol: tolerance.

The mentioned findings suggest that harnessing such endogenous detoxification mechanisms directly within the same species represents a promising strategy for improving Cd tolerance and phytoremediation potential in a broader range of plant systems. While leveraging native physiological pathways and minimizing the need for cross-species genetic manipulation, this approach can also be applied to diverse crops and tree species, thereby expanding its relevance for sustainable phytoremediation across multiple taxa. This expanded relevance aligns with evidence from specific taxa that have already demonstrated notable remediation capacities. For example, some *Brassica* species are effective in phytostabilization, phytovolatilization, and phytoextraction due to their inherent capacity to transport, absorb, and sequester toxic metals into low-activity cellular compartments [26]. Similarly, poplar species offer rapid growth, high biomass, and extensive root systems, making them well-suited for remediation applications. Transgenic poplar plants expressing the ABC transporter gene *YCF1* demonstrate aspects involving potential phytostabilization and phytoattenuation, particularly in heavily contaminated soils where WT plants struggle to survive, while also exhibiting enhanced shoot growth, which may provide additional benefits for biomass production on contaminated sites [24]. Taken together, these examples underscore that integrating species-specific detoxification pathways with targeted ABC transporter manipulation offers a versatile and scalable framework for advancing phytoremediation across diverse plant lineages. Within this overall context, the presented aspects collectively also highlight the potential of functional genetic manipulation and genetically modified plants expressing target ABC transporter genes for phytoremediation-related modulation in Cd-contaminated soils (Figure 4).

Despite considerable progress in this field, several challenges remain—particularly in optimizing gene-host compatibility—which require testing diverse combinations of ABC transporter genes and plant species. Future strategies are expected to integrate the co-expression of metal chelator genes with ABC transporters to develop or enhance phytoremediator plants exhibiting greater efficiency and improved environmental safety in Cd uptake, translocation, and sequestration. Furthermore, while some ABC transporters are recognized for their potential to modulate phytoremediation by improving plant tolerance and Cd accumulation, as some examples presented above, their application is not without complexities and, in certain contexts, can lead to differential outcomes or present significant technical hurdles. Wang et al. [27] demonstrated that ectopic overexpression of *PtoABCG36* from *P. tomentosa* in yeast and *Arabidopsis* conferred increased Cd tolerance to the transgenic lines. These transgenic plants exhibited decreased Cd accumulation in both shoots and roots, whereas WT plants accumulated 1.2 to 1.5 times more Cd. Further measurements indicated reduced Cd uptake in transgenic *Arabidopsis* roots [27]. This mechanism suggests that PtoABCG36 functions as a Cd extrusion pump, actively reducing the metal’s internal content to enhance tolerance [27]. While this might be beneficial for phytostabilization by allowing plants to survive in contaminated soils, it counteracts to some extent the objective of phytoextraction, which relies on maximizing metal uptake into harvestable biomass.

Beyond the specific functional outcomes, the successful deployment of ABC transporters in phytoremediation is also challenged by biological and technical constraints, particularly concerning gene expression. Yazaki et al. [28] investigated the expression of human multidrug resistance-associated protein (hMRP1), an ABC transporter, in transgenic tobacco, observing clear resistance to Cd and vacuolar localization of the transporter. However, in parallel experiments, they reported that despite high hMRP1 mRNA expression in potato, the protein level remained below the detection limit. Similar difficulties were encountered with human MDR1 expression in tobacco, where significant polypeptide accumulation was not observed despite high mRNA levels [28]. These findings underscore that the mere introduction of an ABC transporter gene does not guarantee successful protein expression or the anticipated enhancement of phytoremediation capacity. This necessitates extensive testing of various ABC transporter gene-host plant combinations due to inherent compatibility issues. Therefore, while ABC transporters hold immense promise, their diverse functionalities and expression complexities necessitate a nuanced approach to their deployment in phytoremediation.

## 5. Cross-Kingdom Engineering: A Perspective on Leveraging ABC Transporters for Plant Cadmium Research

A particularly promising frontier involves the strategic harnessing of ABC transporters from diverse microbial organisms and their heterologous expression in plants. This approach expands the genetic toolbox for engineering enhanced Cd tolerance, accumulation, and detoxification, offering a compelling blend of microbial and plant biotechnologies. Relevant work by Song et al. [29] established an early proof-of-concept using the yeast *Saccharomyces cerevisiae*. They identified YCF1, an ABCC subfamily transporter, as a key vacuolar membrane protein responsible for transporting GSH-conjugated heavy metals, including Cd(II) and Pb(II), into the vacuole for detoxification. Their findings demonstrated that a *YCF1*-deletion mutant (*DTY167*) exhibited hypersensitivity to both Pb(II) and Cd(II) compared to WT yeast. Crucially, overexpression of *YCF1* in both yeast cells and *A. thaliana* plants significantly increased resistance to these metals and led to greater accumulation, suggesting its direct utility for phytoremediation [29]. This pioneering study underscored the feasibility of transferring a functionally robust microbial detoxification mechanism into a plant, laying the groundwork for subsequent cross-kingdom genetic manipulations. The broad substrate specificity of YCF1 (Pb and Cd) may be advantageous for multi-metal contaminated sites, though further research could investigate whether transporters with more specific affinities might offer more targeted remediation strategies.

Expanding beyond yeast, recent research has explored more environmentally resilient microbial sources. An investigation involving *Exophiala pisciphila*, a dominant dark septate endophyte naturally thriving in metal-polluted slag heaps. The authors annotated 26 *Ep-ABC* genes, all of which showed metal-dependent transcriptional regulation. Functional characterization through heterologous expression in both metal-sensitive *Saccharomyces cerevisiae* mutants and *A. thaliana* revealed the potent detoxification capabilities of *EpABC2.1*, *EpABC3.1*, and *EpABC4.1*, which are genes from *E. pisciphila* [30]. These transporters not only restored growth in yeast but also significantly enhanced *A. thaliana* tolerance to Pb, Zn, and Cd. Specifically, overexpression of these *EpABC* genes notably promoted plant growth under no Cd supplementation and effectively alleviated the detrimental effects of high Cd stress (1 µmol/L), leading to significant increases in biomass and root length compared to control plants. *EpABC3.1*-overexpressing lines showed the most pronounced improvements, with biomass increases of up to 111.1% under Cd stress [30]. The vacuolar localization inferred for these EpABC transporters suggests their role in enhancing vacuolar compartmentation of toxic ions, a critical detoxification strategy. This work highlights the immense, yet still largely untapped, potential of extremophilic microorganisms as reservoirs of highly efficient metal-tolerance genes for plant bioengineering.

Another compelling avenue for gene sourcing comes from cyanobacteria, as demonstrated very recently by Ruan et al. [31]. Their investigation into *Synechocystis* sp. *PCC 6803* exposed to Cd stress identified a novel ABC transporter, Sll1725, as a core component of its molecular response. Through comprehensive physiological, transcriptomic, and proteomic analyses, Sll1725 was identified as an ABC transporter with a determined Cd efflux function via molecular simulation. Genetic knockdown of *sll1725* in *Synechocystis* resulted in reduced growth and lower photosynthetic efficiency (Fv/Fm), coupled with higher intracellular Cd accumulation, unequivocally confirming its role in mitigating Cd toxicity by efflux. Overexpression of *sll1725* in the aquatic plant *Spirodela polyrhiza* (duckweed) conferred enhanced Cd tolerance [31]. This finding is particularly significant as it introduces an efflux-based detoxification mechanism from a photosynthetic microorganism directly into a plant, offering an alternative or complementary strategy to vacuolar sequestration. While efflux can enhance tolerance by minimizing cellular Cd concentrations, its application in phytoremediation needs careful consideration; for phytoextraction, accumulation is desired, whereas for phytoexclusion in edible crops, reducing internal metal load is paramount.

These studies showcase the transformative potential of leveraging microbial ABC transporters to engineer robust plant responses to Cd contamination. The successful transfer of functional genes from yeast, DSE fungi, and cyanobacteria into diverse plant hosts (*A. thaliana* and *Spirodela polyrhiza*) underscores the conserved molecular mechanisms of ABC transporters across kingdoms and their versatility in adapting to new cellular environments. This rich body of work presents both opportunities and challenges. Future research should prioritize a deeper mechanistic understanding of these microbial ABC transporters, including their precise substrate specificities, transport kinetics, and the energetic costs of their operation within a plant cell. Investigating the potential for synergistic effects by combining different microbial ABC transporters, perhaps targeting distinct subcellular compartments (e.g., vacuolar sequestration via fungal *EpABC* genes and plasma membrane efflux via cyanobacterial *Sll1725*), represents a promising direction. Furthermore, exploring the regulatory networks that govern these genes in their native microbial hosts could provide valuable insights for optimizing their expression and function in genetically modified plants, thereby enhancing control over metal accumulation or exclusion in specific plant tissues. Addressing potential unintended metabolic burdens or pleiotropic effects in recipient plants will also be crucial for real-world applications.

Providing another example of cross-kingdom ABC transporter contribution, the ectopic vacuolar localization of the mammalian ABC transporter hMRP1 in transgenic tobacco conferred clear resistance to Cd [28]. This localization potentially suggests that stable heterologous expression and functional activity of a mammalian transporter might serve as an effective transport engineering strategy in plants. Altogether, insights gained from engineering relevant plant species, including plant models and duckweed with microbial or mammalian ABC transporters, might be translated to a wider array of plants, including economically important crops. Such approaches might enable the development of hyperaccumulating phytoremediation plants or safe crops that limit Cd accumulation in edible tissues.

## 6. Advancing ABC Transporter Research for Strategic Cadmium Management in Plants

Moving forward, it is important to consider the practical applications and challenges of extending the above-mentioned strategies to diverse plant species, highlighting both the opportunities and the considerations for optimizing Cd mitigation in plants alongside relevant functional genetic manipulation aspects. To illustrate and highlight key advances in the field in greater detail, Table 1 provides relevant examples and associated information on major findings regarding ABC transporters in Cd-exposed plants. The progression from initial functional characterizations to insights into specific transporters and their roles in metal detoxification underscores the value of functional genetic studies and complementary approaches (Table 1).

Despite such significant progress, several challenges remain in harnessing ABC transporters for comprehensive Cd mitigation:

- Redundancy and Specificity: The possible redundancy within the ABC transporter superfamily means that manipulating a single gene may not always yield a strong phenotype due to compensatory mechanisms. Moreover, the broad substrate specificity of some ABC transporters requires careful consideration to avoid unintended effects on essential nutrient homeostasis or the accumulation of other undesirable compounds (Table 1).
- Tissue-Specific, Developmental Regulation, and Tissue-Specific Promoters for Spatial Control: Achieving precise control over Cd accumulation requires tissue-specific and developmentally regulated gene expression. For example, promoting Cd efflux from roots while simultaneously enhancing its sequestration in non-edible leaves, or limiting its transport into reproductive organs, demands sophisticated genetic engineering approaches. Within this context, an important aspect in the genetic manipulation of ABC transporters for Cd mitigation involves the use of tissue-specific promoters. They often align with the plant’s developmental stages and can be categorized according to target tissues, such as roots, stems, leaves, flowers, or seeds. By combining regulatory elements through synthetic biology, these promoters enable fine-tuned modulation of metabolic pathways and stress responses, offering versatile tools for improving plant performance and designing tailored genetic interventions [32]. As evidenced by current studies (Table 1), several genetic constructs have relied on constitutive promoters which drive ubiquitous gene expression across multiple plant organs. While effective for functional characterization, such expression pattern may eventually cause unintended pleiotropic effects, including altered growth, metabolic imbalances, or energy costs in non-target tissues. Thus, future studies might leverage tissue-specific promoters to achieve spatially precise expression of ABC transporters, thereby optimizing plant tolerance while minimizing unintended effects in non-target tissues and providing a refined strategy for targeted Cd detoxification.
- Multi-gene Engineering: A promising avenue for enhancing plant tolerance to multiple toxic elements lies in multigene engineering, combining ABC transporters with genes involved in relevant pathways. A compelling example of this approach was recently demonstrated in rice, where the co-overexpression of *OsPCS1*, *OsABCC1*, and *OsHMA3* led to dramatic reductions in As and Cd concentrations in the grain, without any detrimental effects on plant growth, reproduction, or essential mineral nutrient content [19]. Furthermore, co-expression of *AtMRP7* with *AtPCS1* was shown to alleviate the Cd-hypersensitivity caused by *AtPCS1* overexpression, as the *AtMRP7/AtPCS1* double-transformants exhibit fewer Cd-induced necrotic lesions despite similar shoot Cd levels in tobacco. This indicates that AtMRP7 enhances detoxification, likely by promoting the removal of Cd or PC–Cd complexes from the cytosol, thereby restoring the balance required for effective Cd sequestration [13]. Thus, incorporating approaches in which broad-specificity ABC transporters are co-engineered with metal-related genes might maximize detoxification and accumulation efficiency in different plant systems. This further underscores the value of strategically designed multigene approaches to overcome potential limitations of single-gene manipulation—such as redundancy or limited substrate specificity—while minimizing pleiotropic effects.
- Complex Interactions: The interplay between ABC transporters and other metal homeostasis components needs further elucidation. Understanding these complex networks is crucial for designing more effective and ABC-targeted interventions.
- Structural and Holistic Functional Characterization: Several studies have functionally analyzed ABC transporters through in vitro assays, ectopic genetic manipulation, interspecific strategies, or heterologous expression systems in yeast and other model organisms (Table 1), providing valuable insight into their potential roles in Cd transport. However, these approaches remain largely indirect, leaving many mechanistic aspects unresolved, and understanding these proteins requires a holistic approach that integrates structural, biochemical, and physiological analyses. To truly elucidate transporter function, future research might move toward protein-level structural and biochemical analyses, focusing on substrate-binding dynamics, ATP hydrolysis mechanisms, and conformational transitions that define transport directionality. Integrating multiple strategies such as protein modeling, and site-directed mutagenesis with in planta functional assays, for instance, will be relevant to bridge current genetic knowledge with precise molecular understanding. Such studies will ultimately clarify how ABC transporter structure dictates function, providing a more robust foundation for rational engineering aimed at Cd detoxification in plants.
- Metabolic Fine-Tuning of ABC Transporters for Cd Mitigation and Quality Optimization: Recent insights into CsABCG11.2 have underscored the need to view Cd tolerance not merely as a detoxification mechanism but as a metabolic equilibrium involving amino acid and nitrogen fluxes [8]. Future studies might focus on the fine-tuning of strategic ABC transporters, such as modifying CsABCG11.2 activity or substrate affinity rather than relying solely on gene silencing. Integrating transporter function into breeding and metabolic engineering frameworks could enable the development of genotypes or cultivars with balanced metabolite profiles, improved nutrient efficiency, regulated nitrogen metabolism, and minimized Cd accumulation, while preserving agronomic performance and commercial quality.
- Multi-stress and Combined Stress Studies: While several ABC-related investigations have focused on single-metal or single-stress scenarios (Table 1), real-world environments often present multiple co-occurring stresses, such as combinations of heavy metals or other abiotic and biotic stressors. Understanding how ABC transporters respond under such combined conditions is critical for developing robust plant genotypes capable of coping with multifactorial stress environments. Future research might explore synergistic or antagonistic effects on transporter activity, metal sequestration, and overall plant physiology, potentially guiding multi-targeted genetic interventions in the research context covering ABC proteins and plant genetic engineering tools.
- Multi-omic Integration: Building on this framework, our laboratories have focused on integrating multi-omic approaches in plants to unravel the complex regulation and interactions of molecules, including ABC transporters, under Cd stress [1,33]. Further research regarding this research strategy through complementary genetic engineering strategies is highly relevant for functional validation and potential translational applications.
- Translational Research: In addition to the various aspects mentioned in the topics above in previous sections, we emphasize that translating promising laboratory findings from model plants (like *Arabidopsis*) to high-biomass crops (like poplar, *Brassica* species) and staple food crops (like rice and wheat) under field conditions is a significant aspect to be addressed. Factors such as genetic background, environmental variability, and complex soil chemistry must be thoroughly investigated. Several of these translational dimensions have already been integrated throughout the previous sections, including, for instance, aspects of pleiotropic and field-relevant physiological trade-offs, and the recognition of constraints associated with single-gene modifications. Moreover, Figure 1, Figure 2, Figure 3 and Figure 4 and Table 1 consolidate these points by highlighting laboratory procedures with potential agronomic performance, soil–metal interactions, and implications for potential deployment under realistic cultivation scenarios, thereby outlining a structured progression from mechanistic insights to practical application. Together, these research aspects establish a coherent framework for translating ABC transporter research into field-applicable strategies across multiple plant species and environmental conditions upon Cd exposure.

### Bibliometric Analysis of Research Trends

**Figure 5:**
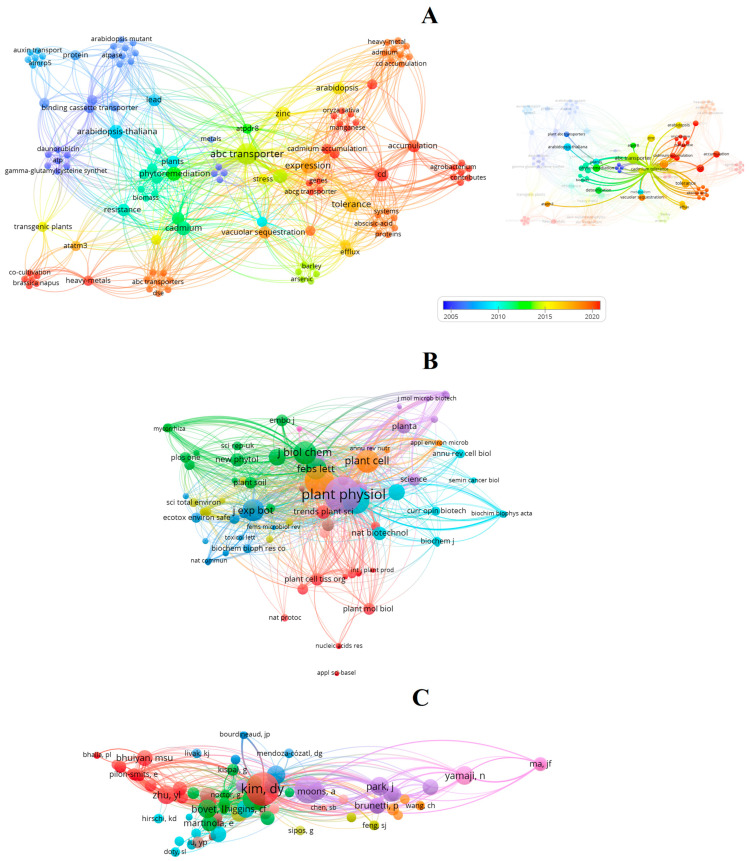
Bibliometric analysis of research on ABC transporters in Cd-exposed plants within the functional genetic manipulation framework. (**A**) Co-occurrence network of keywords extracted from the analyzed dataset, visualized using VOSviewer. (**B**) Co-citation network of cited sources. (C) Co-citation network of cited authors. Each node (sphere) represents a keyword, journal, or author, and its size is proportional to the frequency or number of citations. The color gradient in (**A**) indicates the average publication year (from blue = earlier studies to red = recent studies), while the different colors in (**B**,**C**) correspond to distinct clusters of journals or authors sharing co-citation relationships. The connecting lines represent the strength of association between elements, with thicker links denoting stronger co-occurrence or co-citation relationships.

We additionally conducted a bibliometric assessment based on an international database to provide an integrated perspective on the main scientific themes and focal points associated with ABC transporters in the contexts of functional genetic manipulation and Cd-related plant studies. Detailed information on the methodology is available in Supplementary Material S1. By employing the VOSviewer software (version 1.6.20; Leiden University, The Netherlands) [34] and systematically analyzing the retrieved publications, we constructed Figure 5A–C, which presents multiple network visualizations highlighting distinct research dimensions, as well as emerging and influential trends within this field.

In the co-occurrence network of keywords (Figure 5A), major thematic clusters can be identified. The first cluster (blue) encompasses early foundational studies focused on *A. thaliana* mutants, ATPases, and binding cassette transporters, mainly between 2005 and 2010. These investigations laid the biochemical and molecular basis for understanding ABC transporter function and relevant Cd detoxification mechanisms. The green–yellow cluster represents a transition phase emphasizing phytoremediation, resistance, and vacuolar sequestration, while the red–orange cluster includes more recent research (2015–2020) exploring cadmium accumulation, tolerance, *Agrobacterium*-mediated transformation, and expression studies in various plant systems. Although this progression reveals a steady diversification of the field, the analysis also highlights that the majority of research remains concentrated in model species, such as *A. thaliana* and *O. sativa*. Studies addressing socioeconomically relevant crop species remain comparatively limited. This limited taxonomic scope restricts the translational impact of findings and underlines the need for expanding experimental approaches toward crops of agronomic importance, particularly under field conditions.

In parallel, the co-citation network of cited sources (Figure 5B) reveals that journals such as Plant Physiology, Plant Cell, and the Journal of Biological Chemistry act as major hubs within the literature. Strong interconnections were also observed with Plant Molecular Biology, Plant Biotechnology Journal, and New Phytologist, underscoring the cross-disciplinary scope of ABC transporter research, which bridges molecular biology, biochemistry, and environmental physiology. Nonetheless, the predominance of plant physiology-oriented journals over those emphasizing biotechnology or applied genetic engineering may suggest that most functional validation studies continue to focus on mechanistic understanding rather than translational applications or applied genome-editing innovations.

The co-citation analysis of authors (Figure 5C) further supports this interpretation. Some frequently listed co-cited authors—such as Kim DY, Moons A, Yamaji N, and Ma JF—have contributed to elucidating heavy metal transport, vacuolar sequestration, or gene function characterization in different plant species. Yet, despite these influential contributions, there is still limited evidence of large-scale integration of new-generation functional tools, such as CRISPR/Cas-based genome editing or synthetic promoter design, within ABC transporter research under Cd stress.

When analyzed in conjunction with data summarized in Table 1, the bibliometric findings suggest that many studies categorized under genetic manipulation focus primarily on relevant functional evaluation strategies for both phytoremediation and crop safety purposes. Network analyses also highlight a dynamic coverage involving both studies addressing phytoremediation potential and those centered on crop safety, revealing that both directions increasingly share molecular frameworks, with ABC transporters occupying a central position in the network (Figure 5A). The co-occurrence of terms such as phytoremediation, cadmium tolerance, vacuolar sequestration, and ABC transporter (Figure 5A) reflects an integrated research trend aimed not only at modulating Cd uptake and detoxification capacities but also at minimizing the toxic metals in relevant plant crop organs.

Overall, the integrated bibliometric analysis (Figure 5) indicates both the conceptual maturity and current limitations of the field. While the mechanistic understanding of ABC transporters in Cd detoxification has been investigated, there remains significant room for innovation—particularly in extending molecular discoveries to crop improvement programs, considering multi-omics integrations, incorporating CRISPR-based precision editing, multiple prominent plant species and experimental settings, synthetic regulatory systems, and evaluating the resulting genotypes under realistic agricultural conditions. These directions are relevant to ensure that advances in ABC transporter research contribute effectively to sustainable and economically viable strategies for mitigating Cd stress in plants. Furthermore, expanding future research to include genuine genetic engineering tools could provide deeper insights and greater applied potential, provided that such strategies are developed with careful consideration of agronomic viability, relevant ecological parameters, and environmental sustainability. In this context, functional genetic manipulation has emerged as a critical approach to balancing these multiple objectives. Overexpression, silencing, and heterologous expression of specific ABC transporter genes have been strategically explored to create genotypes capable of both efficient remediation and reduced metal accumulation in harvestable tissues (Table 1; Figure 5). Such strategies mark a conceptual shift from purely unidirectional models toward bioengineered plants that combine environmental detoxification with food safety assurance.

## 7. Concluding Remarks and Additional Future Directions

ABC transporters are key components of plant defense mechanisms against Cd stress. Their diverse localization and substrate specificities enable plants to efflux Cd from cells, sequester it into vacuoles, or transport it across different tissues. Through precise and functional genetic manipulation, these transporters can be harnessed to enhance Cd removal from contaminated environments via phytoremediation or to safeguard food safety by preventing Cd accumulation in edible plant parts. Balancing or exploring these two objectives requires a nuanced understanding of each transporter’s function, tissue-specific expression, and potential pleiotropic effects, along with more holistic mechanistic insights. Future research integrating multi-omics approaches with advanced gene-editing technologies will likely unlock the full potential of ABC transporters, offering sustainable solutions to global Cd pollution while protecting agricultural productivity and human health.

As we previously discussed [1], increasing Cd tolerance does not necessarily improve agronomic performance, highlighting the importance of assessing trade-offs between detoxification capacity and growth or yield in crop species. Several studies on genetic manipulation have relied on transient or stable gene function alterations without direct genome engineering (Figure 5; Table 1). These approaches remain highly relevant, particularly when they address key phenotypic outcomes such as biomass accumulation, metal partitioning, and the balance between tolerance and productivity. Bibliometric analysis (Figure 5) further indicates that, although classical model species-based investigations remain central to mechanistic studies, an increasing number of investigations—albeit still limited—extend these insights to other plant species, bridging fundamental plant physiology with applied biotechnology. This trend highlights that functional genetic manipulation of ABC transporters could evolve from narrowly targeted applications to a pivotal approach for enhancing sustainable crop production upon Cd exposure.

## Figures and Tables

**Table 1:** Examples of studies on ABC transporters and genetic manipulation in Cd-exposed plants.

Plant Species (Target of Genetic Manipulation)	ABC Transporter Genes/Proteins	Approaches (Genetic Manipulation)	Cd Exposure/Treatment Conditions	Examples of Observed Outcomes	References
*Arabidopsis thaliana*	*AtABCC3*	Induced Overexpression (OE) (*AtABCC3ox*) using β-oestradiol	30–90 μM CdSO_4_; 9 d exposure (seedlings); 10 μM β-oestradiol induction; leaf protoplasts analyzed.	Increased Cd tolerance; Increased vacuolar Cd sequestration and concomitant decrease in cytosolic Cd; Activity dependent on Phytochelatins (PCs).	[7]
*Arabidopsis thaliana*	*AtMRP6* (also known as *AtABCC6*)	Knockout (KO) (Transfer DNA (T-DNA) insertion, *Atmrp6.1*, *Atmrp6.2*)	1–5 μM CdSO_4_; 21 d exposure; Seedlings analyzed.	Increased Cd sensitivity in seedlings; Significantly lower rosette-leaves Fresh weight (FW) in mutants compared to Wild-type (WT) after Cd treatment.	[14]
*Arabidopsis thaliana*	*AtATM3*	OE (35S::*AtATM3*)	40 μM CdCl_2_ (2–3 wks, seedlings); 100 μM CdCl_2_ (24 h root treatment).	Enhanced Cd resistance (1.5–2-fold higher FW); Increased shoot Cd content; Resistance mechanism requires Glutathione (GSH).	[10]
*Arabidopsis thaliana*	*AtPDR8*	OE (35S::*AtPDR8*)	40 μM CdCl_2_ (2–3 wks, seedlings); 0.1 μM ^109^CdCl_2_ (12 h uptake).	Enhanced Cd resistance (1.5–1.8-fold higher FW); Lower shoot and root Cd content; Higher Cd efflux rate (functions as a Plasma membrane (PM) extrusion pump).	[12]
*Arabidopsis thaliana*	*AtABCC1*	OE (35S::*AtABCC1*)	40 or 60 μM CdCl_2_; 3 wks exposure (seedlings).	Enhanced Cd tolerance (higher shoot FW); Increased Cd accumulation in shoot, root, and total Cd content per plant.	[11]
*Nicotiana tabacum* var. Xanthi	*AtMRP7*	OE (Heterologous expression under CaMV35S promoter)	25 mM CdCl_2_ (6 d); 5 mM CdCl_2_ (3 d).	Increased Cd tolerance (higher shoot Dry matter (DM) yield); Increased Cd storage in leaf vacuoles (2–3-fold higher); Restricted Root-to-shoot concentration ratio (R/S) Cd translocation. *AtMRP7* localized at PM and Tonoplast.	[13]
*Triticum aestivum*	*TaABCC13*	RNA interference (RNAi) (Constitutive expression)	50 μM CdCl_2_; 7 d exposure (seedlings).	Increased Cd sensitivity (significantly lower shoot biomass); Reduced Cd uptake in roots and shoots.	[21]
*Oryza sativa*	*OsABCG48* (ABCG transporter)	OE; Transgenic expression (*Agrobacterium*)	2 μM CdCl_2_ (5 d); 2 μM Cd (12 h after 28 d growth).	Enhanced Cd tolerance; Less root Cd accumulation than WT; Grew 3- to 4-fold more lateral roots under Cd stress.	[15]
*Oryza sativa* cv. Nipponbare	*OsABCG36*	KO (CRISPR/Cas9, *osabcg36-1*, *osabcg36-2*)	2 μM Cd (5 d seedlings); 5 μM CdSO_4_ (14 d); 20 μM Cd (8 h).	Enhanced Cd sensitivity (inhibited root growth); Increased root Cd concentration; Functions as a PM-localized efflux transporter.	[16]
*Oryza sativa*	*OsABCC1* (Co-OE with *OsPCS1*, *OsHMA3*)	Co-OE (under *OsActin1 promoter*)	Paddy soil environment; Cd analyzed in grain.	Decreased Cd concentration in grain by 98% compared with non-transgenic control.	[19]
*Arabidopsis thaliana*	*CsABCG11.2*	OE (35S::*CsABCG11.2*)	40 μM or 100 μM Cd (CdCl_2_); 2 wks plate/1 mo hydroponics; +/−20 μM Thea.	Increased Cd sensitivity (lethal dose); Enhanced Cd accumulation in shoots (76.5% translocation); Exogenous Thea mitigated toxicity.	[8]
*Camellia sinensis*	*CsABCG11.2*	Knockdown (KD) (Virus-Induced Gene Silencing (VIGS), TRV-based)	40 μM Cd (CdCl_2_) +/−50 μM Thea; 3 wks.	Reduced Cd accumulation in Young Leaves (YL); Confirmed role in Cd translocation from root to shoot.	[8]
*Oryza sativa*	*OsPDR20*	RNAi/KD, KO (T-DNA)	Hydroponics: 2 or 10 μM Cd; 16 d. Field: 0.40 mg/kg Cd soil.	Increased Cd accumulation (1.91–2.97 folds in brown rice); Compromised growth/sensitivity; Suggests *OsPDR20* functions to reduce Cd accumulation.	[18]
*Oryza sativa*	*OsABCC1*	KO (T-DNA insertion)	Cd: Low/High conc.	No effect on Cd toxicity (Primary study focus was Arsenic (As)).	[20]
*Oryza sativa*	*OsABCG43*	OE (Under maize Ubi promoter); KO (CRISPR/Cas9)	Hydroponics: 2.0, 5.0, or 30 μM CdCl_2_ (10–20 d); Field conditions.	Functions as a PM-localized Cd Importer. OE lines showed enhanced Cd accumulation (up to 3.0-fold in xylem sap); Resulted in Phytotoxicity and enhanced Cd sensitivity.	[4]
*Oryza sativa* (cv. Nipponbare)	*OsABCC9*	KO (CRISPR/Cas9)	Hydroponics: 5 or 10 μM Cd (CdSO_4_); 12 d. Field: 2.0 mg/kg Cd soil.	KO lines exhibited enhanced Cd sensitivity (reduced root/shoot Dry weight (DW)); Accumulated more Cd; Sharply increased Cd concentration in grain (2–3 fold); Tonoplast-localized transporter mediating vacuolar sequestration of Cd.	[17]
*Triticum turgidum* subsp. *durum*	*TaABCG2-5B*	KO (TILLING/EMS, *ΔTaabcg2-5B*)	2 mM Cd^2+^ (5 d).	≈32% Decrease in Cd accumulation in root; Confirms function as a PM-localized Cd Importer.	[9]
*Triticum turgidum* subsp. *durum*	*TaABCG2-5B*	OE (UBI promoter, OE-*TaABCG2-5B*-16)	2 mM Cd^2+^ (5 d).	≈106% Increase in Cd accumulation in root; Confirms role as a PM-localized Cd Importer.	[9]
*Brassica juncea*	YCF1	OE; *Agrobacterium*-based transformation	0.15 M Cd(II) (CdCl_2_); 7 d (tolerance); 11 d (accumulation).	Enhanced Cd tolerance (1.3- to 1.6-fold higher FW than WT); Significantly increased accumulation of Cd(II) in shoot tissues.	[22]
*Brassica juncea*	*AtATM3*	OE; Transgenic expression (*Agrobacterium*)	0.15 M Cd(II) (CdCl_2_); 7 d (tolerance); 9–11 d (accumulation).	Enhanced Cd tolerance (2.2- to 2.3-fold higher FW than WT); Increased Cd accumulation in shoot; Upregulated *BjGSHII* and *BjPCS1* transcripts.	[25]
*Populus alba* × *P. tremula* var. *glandulosa* (BH)	*ScYCF1*	OE; Transgenic expression (*Agrobacterium*)	Tailing soil (43 mg kg^−1^ Cd) 2 wks; 1:1 Tailing soil 2 mos; Hydroponics: 1 ppm Cd, 4 wks.	Enhanced tolerance; Accumulated up to 5-fold more Cd in shoot than WT; Increased root system size; Increased accumulation of Cd, Zinc (Zn), and Lead (Pb) in root.	[24]
*Brassica napus* cv. BARI Sarisha-8	*AtATM3*	OE; Transgenic expression (*Agrobacterium*)	0.15 M CdCl_2_ (7 d); 1/2 MS medium.	Enhanced Cd tolerance (1.4- to 1.7-fold higher FW than WT); Also showed increased Pb tolerance.	[26]
*Arabidopsis thaliana*	*PtoABCG36* (ABCG transporter)	OE; Transgenic expression (35S promoter)	40 or 60 μM CdCl_2_ (2 wks); 100 μM CdCl_2_ (24 h, accumulation/Non-invasive micro-test (NMT)).	Enhanced Cd tolerance; Decreased Cd accumulation in shoot and root; Confirms function as a Cd extrusion pump (decreased net Cd^2+^ influx).	[27]
*Arabidopsis thaliana*	*EpABC2.1*; *EpABC3.1*; *EpABC4.1* (ABC transporters)	OE; Heterologous expression from *E. pisciphila*	0, 0.1, or 1 μM Cd (20 d); 0, 0.2, or 0.4 mmol/kg Cd (25 d).	Enhanced Cd tolerance; Promoted Cd accumulation; Suggests detoxification via vacuolar compartmentalization.	[30]
*Spirodela polyrhiza* (Duckweed)	*sll1725* (Type IV ABC transporter)	OE; Transgenic expression (UBI promoter; *Agrobacterium*)	5 mg L^−1^ Cd^2+^; 5 d.	Enhanced Cd tolerance; Significantly higher Wet weight (WW) and DW than WT.	[31]
*Nicotiana tabacum*	*hMRP1* (MRP subfamily of ABC transporter)	OE; Transgenic expression (*Agrobacterium*)	0–100 μM CdCl_2_ (10 d, cultured cells); 0–480 μM Cd (14 d, seedlings).	Conferred clear resistance/tolerance to Cd (e.g., maintained chlorophyll content, greater FW/root length); *hMRP1* localized at the vacuolar membrane.	[28]

The following abbreviations are used in the Table: ABC: ATP-binding cassette; ABCG: ATP-binding cassette G subfamily; *Agrobacterium*: *Agrobacterium tumefaciens* (used for transformation); As: Arsenic; *AtABCC1*: *Arabidopsis thaliana* ABC C-type transporter 1; *AtABCC3*: *Arabidopsis thaliana* ABC C-type transporter 3; *AtABCC3ox*: *AtABCC3* overexpressors; *AtATM3*: *Arabidopsis thaliana* ABC Transporter of the Mitochondria 3; *AtMRP6*: *Arabidopsis thaliana* Multidrug resistance-associated Protein 6 (also known as *AtABCC6*); *AtMRP7*: *Arabidopsis thaliana* Multidrug resistance-associated Protein 7; *AtPDR8*: *Arabidopsis thaliana* Pleiotropic Drug Resistance 8; *Atmrp6.1*: *AtMRP6* T-DNA insertion knockout mutant 6.1; *Atmrp6.2*: *AtMRP6* T-DNA insertion knockout mutant 6.2; BH: Bonghwa control plant (*Populus alba* × *P. tremula* var. *glandulosa* non-transgenic clone); *BjGSHII*: *Brassica juncea* glutathione synthetase II; *BjPCS1*: *Brassica juncea* phytochelatin synthase 1; Cd: Cadmium; Cd^2+^: Divalent Cadmium ion; Cd(II): Divalent Cadmium ion; CdCl_2_: Cadmium chloride; CdSO_4_: Cadmium sulfate; CaMV35S promoter: Cauliflower Mosaic Virus 35S promoter; Co-OE: Co-overexpression; Constitutive expression: Refers to the use of the CaMV 35S promoter to drive RNAi expression ubiquitously; CRISPR/Cas9: Clustered Regularly Interspaced Short Palindromic Repeats/Cas9 endonuclease system; *CsABCG11.2*: *Camellia sinensis* ATP-Binding Cassette G 11.2; cytosolic Cd: Cytosolic Cadmium; d: Days; DM: Dry matter; DW: Dry weight; *ΔTaabcg2-5B*: Loss-of-function mutant of *TaABCG2-5B*; efflux transporter: Transporter mediating export out of the root cells; *EpABC2.1*: *Exophiala pisciphila* ABC transporter 2.1; *EpABC3.1*: *Exophiala pisciphila* ABC transporter 3.1; *EpABC4.1*: *Exophiala pisciphila* ABC transporter 4.1; FW: Fresh weight; GSH: Glutathione; h: Hours; *hMRP1*: human multidrug resistance-associated protein 1; Importer: Transporter facilitating substance transport into the cytoplasm; KD: Knockdown; KO: Knockout; M: Molar; mg L^−1^: Milligrams per liter (concentration unit); mg/kg: Milligram per kilogram; mg·kg^−1^: Milligram per kilogram; mM: Millimolar; mmol/kg: millimole per kilogram (concentration unit for soil treatment); mo: Month(s); MRP: Multi-drug resistance-associated protein; MS: Murashige and Skoog medium; NMT: Non-invasive micro-test (technique for flux measurement); OE: Overexpression; *OsABCC1*: *Oryza sativa* ABCC transporter 1; *OsABCC9*: *Oryza sativa* ABCC transporter 9; *OsABCG36*: *Oryza sativa* ABC G-type transporter 36; *OsABCG43*: *Oryza sativa* ABC G-type transporter 43; *OsABCG48*: *Oryza sativa* ABCG48; *OsHMA3*: *Oryza sativa* Heavy Metal ATPase 3; *OsPCS1*: *Oryza sativa* Phytochelatin Synthase 1; *OsPDR20*: *Oryza sativa* Pleiotropic Drug Resistance 20; *OsActin1 promoter*: Rice Actin 1 promoter (used to drive expression); *osabcg36-1*: *OsABCG36* knockout line 1; *osabcg36-2*: *OsABCG36* knockout line 2; Pb: Lead; PCs: Phytochelatins; Phytotoxicity: Toxicity resulting in damage to the plant (e.g., inhibited growth, accelerated senescence, lesions or spots); PM: Plasma membrane; ppm: parts per million; *PtoABCG36*: *Populus tomentosa* ABCG36; R/S: Root-to-shoot concentration ratio; RNAi: RNA interference; *ScYCF1*: *Saccharomyces cerevisiae* Cadmium factor 1; *sll1725*: Gene Sll1725 (ABC transporter from *Synechocystis* sp. PCC 6803); *TaABCC13*: *Triticum aestivum* ABCC transporter 13; *TaABCG2-5B*: *Triticum aestivum* ABC G-type transporter 2-5B; T-DNA: Transfer DNA; Thea: Theanine; TILLING/EMS: Targeting Induced Local Lesions in Genomes/Ethyl Methane Sulfonate (mutation induction method); TRV-based: Tobacco rattle virus-based system; *Triticum turgidum* subsp. *durum*: Durum wheat subspecies used in the study; Ubi promoter: Maize Ubiquitin promoter; UBI promoter: Ubiquitin promoter; vacuolar Cd: Vacuolar Cadmium; VIGS: Virus-Induced Gene Silencing; wks: Weeks; WT: Wild-type; WW: Wet weight; YCF1: Yeast Cadmium Factor 1; YL: Young Leaves; Zn: Zinc; ^109^CdCl_2_: Radioactive Cadmium chloride; 35S::*AtABCC1*: *AtABCC1* gene expression driven by the Cauliflower Mosaic Virus 35S promoter; 35S::*AtATM3*: *AtATM3* gene expression driven by the Cauliflower Mosaic Virus 35S promoter; 35S::*AtPDR8*: *AtPDR8* gene expression driven by the Cauliflower Mosaic Virus 35S promoter; 35S::*CsABCG11.2*: *CsABCG11.2* gene expression driven by the Cauliflower Mosaic Virus 35S promoter; β-oestradiol: Inducer chemical for overexpression; μM: Micromolar.

## Data Availability

No new data were created or analyzed in this study. Data sharing is not applicable to this article.

## Supplementary Materials

The following supporting information can be downloaded at: https://www.mdpi.com/article/10.3390/ijms262311662/s1.
