# Supplementary material for: Functional Genetic Frontiers in Plant ABC Transporters: Avenues Toward Cadmium Management
Source: Int J Mol Sci. 2025 Dec 2;26(23):11662. doi: 10.3390/ijms262311662 (PMC12691841; doi:10.3390/ijms262311662)
Supplement: Supplementary file 1 [file ijms-26-11662-s001.zip › ijms-3953586-supplementary.pdf]

Supplementary file 1

Functional Genetic Frontiers in Plant ABC Transporters: Avenues Toward Cadmium Management

Deyvid Novaes Marques [1]*, Chase M. Mason[2]

[1] Department of Genetics, Luiz de Queiroz College of Agriculture (ESALQ), University of São Paulo (USP), Piracicaba, São Paulo (SP), Brazil
[2] Department of Biology, University of British Columbia Okanagan, Kelowna, British Columbia, Canada; chase.mason@ubc.ca

* Correspondence: deyvidnovaes@gmail.com

Bibliometric Search and Analysis of Functional Genetic Manipulation regarding ABC Transporters in Cadmium-Exposed Plants

To construct a bibliometric dataset focusing on the intersection between ABC transporters, cadmium (Cd) exposure, and functional genetic manipulation in plants, a systematic search was conducted in the Web of Science Core Collection. The search strategy was carefully designed to retrieve publication abstracts that explicitly addressed ATP-binding cassette (ABC) transporters in the context of Cd stress and functional genetic modification in plants. The query combined terms referring to ABC transporters ("ABC," "ABC transporter," or "ATP-binding cassette") with plant-related descriptors (including "plant," "Arabidopsis," "rice," "wheat," "tobacco," and "poplar") and genetic manipulation approaches such as "transgenic," "overexpression," "silencing," "RNAi," "heterologous expression," and "mutant." These terms were further associated with "cadmium" or its chemical symbol "Cd" to ensure the retrieval of studies explicitly addressing Cd exposure.

This comprehensive combination of descriptors ensured the inclusion of experimental studies that investigated ABC transporter genes in Cd-exposed plants through both transient expression systems (such as RNA interference or heterologous expression) and stable genetic modifications (including mutant generation or transgenic overexpression). The focus was placed on research exploring molecular and physiological mechanisms underlying Cd uptake, transport, detoxification, and tolerance mediated by ABC transporters, thereby reflecting genuine cases of functional genetic manipulation.

After retrieval, all records were carefully curated and manually filtered. Review articles were excluded, and only original research papers were retained. Studies that did not focus on ABC transporters, as well as those unrelated to ABC transporter-focused functional genetic manipulation or plant Cd exposure—even when other metal-related regulatory genes were discussed—were also removed to ensure thematic precision and consistency across the dataset. Consequently, the final dataset comprised high-quality research specifically centered on the functional roles of ABC transporters in Cd-exposed plants.

The curated and filtered records were then exported from the Web of Science Core Collection and analyzed using VOSviewer (version 1.6.20, Leiden University, The Netherlands) [1]. VOSviewer was employed to construct bibliometric maps, visualize term co-occurrence networks, and identify conceptual clusters, allowing a detailed interpretation of how ABC transporter research integrates both phytoremediation and crop safety considerations within the broader framework addressing plant exposure and responses to Cd.

Reference

[1] van Eck, N.J.; Waltman, L. Software survey: VOSviewer, a computer program for bibliometric mapping. Scientometrics 2010, 84(2), 523-538. https://doi.org/10.1007/s11192-009-0146-3
